# Prognostic Value of Automated Bone Scan Index (aBSI) in Patients with mCRPC Undergoing Three vs. Six Cycles of ^223^Ra Therapy

**DOI:** 10.3390/diagnostics15162007

**Published:** 2025-08-11

**Authors:** Sophie C. Siegmund, Harun Ilhan, Antonia Gerull, Andrei Todica, Marcus Unterrainer, Astrid Delker, Franz Josef Gildehaus, Can D. Aydogdu, Christian G. Stief, Rudolf A. Werner, Lena M. Unterrainer, Mathias J. Zacherl

**Affiliations:** 1 Department of Nuclear Medicine, LMU University Hospital, LMU Munich, 81377 Munich, Germany; 2 Bavarian Cancer Research Center (BZKF), Partner Site, 81377 Munich, Germany; 3 Die Radiologie, 81925 Munich, Germany; 4 Kinderarztpraxis am Arabellapark, 81925 Munich, Germany; 5 Department of Urology, LMU University Hospital, LMU Munich, 81377 Munich, Germany; 6 The Russell H Morgan Department of Radiology and Radiological Sciences, Division of Nuclear Medicine, Johns Hopkins School of Medicine, Baltimore, MD 21287, USA; 7 Ahmanson Translational Theranostics Division, Department of Molecular and Medical Pharmacology, University of California Los Angeles UCLA, Los Angeles, CA 90095, USA

**Keywords:** ^223^Radium-therapy, mCRPC, bone metastases, overall survival, disease burden, cycles, aBSI

## Abstract

**Background/Objectives**: In patients with metastatic castration-resistant prostate cancer (mCRPC) and osseous metastases only, ^223^Radium therapy represents a valuable therapeutic option. Bone scintigraphy (BS) is typically performed to assess metastasis load, with the BS-derived automated bone scan index (aBSI) used for response assessment. This study aimed to evaluate the prognostic value of aBSI in patients receiving three or six cycles of ^223^Ra therapy. **Methods**: We included patients that were diagnosed with extensive osseous tumor load on BS, had no visceral or nodal metastases, had undergone ^223^Ra therapy. The aBSI prior to and following three or six cycles of therapy, total tumor volume (TTV), SUV_max_, and overall survival were analyzed. **Results**: This study included 49 mCRPC patients (mean age: 70 ± 9 years) with 42 (85.7%) receiving six and 7 (14.3%) receiving three cycles. After three cycles, the mean aBSI (*p* = 0.369), TTV (*p* = 0.902), and SUV_max_ (*p* = 0.149) remained unchanged. After six cycles, the mean aBSI (*p* = 0.247) and TTV (*p* = 0.784) were unchanged, while SUV_max_ decreased significantly (*p* = 0.001). The aBSI did not significantly correlate with the mean aBSI (six cycles: χ^2^ = 1.823, *p* = 0.177; three cycles: χ^2^ = 0.308, *p* = 0.579). **Conclusions**: Although quantitative changes in TTV and aBSI did not significantly correlate with each other, their respective absolute values consistently indicated stable disease burden under therapy. This highlights its potential as a useful tool for monitoring disease burden while indicating that aBSI alone is insufficient for predicting overall survival.

## 1. Introduction

In advanced prostate cancer, resistance to androgen deprivation therapy (ADT) necessitates alternative treatment options, particularly for patients who progress to metastatic castration-resistant prostate cancer (mCRPC) [1,2]. In this setting, the selection of therapy depends largely on the pattern of metastatic spread [3,4]. Bone scintigraphy (BS) using ^99m^Tc-phosphonate is commonly employed to evaluate the extent of osseous tumor burden [5]. Patients with osseous metastases, but without visceral or nodal metastases, may be eligible for treatment with ^223^Radium (^223^Ra) dichloride [3,6]. This alpha particle-emitting radiopharmaceutical selectively targets bones by mimicking calcium and inducing double-strand DNA breaks in tumor cells within the bones [7]. The international phase III ALSYMCA trial demonstrated that ^223^Ra significantly prolongs overall survival (OS) and reduces the risk of symptomatic skeletal events compared to placebo. Consequently, ^223^Ra was approved by the FDA for treating patients with mCRPC, symptomatic bone metastases, and no visceral metastases [8]. Automated bone scan index (aBSI), a quantitative parameter derived from BS, has emerged as a promising prognostic biomarker to assess disease burden and monitor therapeutic response in mCRPC patients undergoing ^223^Ra therapy. Changes in the aBSI have been shown to correlate with overall survival [9,10,11].

The objective of this study was to evaluate the prognostic value of the aBSI in assessing treatment response in patients receiving three or six cycles of ^223^Ra therapy.

## 2. Materials and Methods

### 2.1. Patients

We included patients that were diagnosed with an extensive osseous tumor load on BS, had no visceral or nodal metastases, and had undergone ^223^Ra therapy. This analysis was performed in compliance with the principles of the Declaration of Helsinki and was approved by the institutional ethics board of the LMU Munich (230-15, approved date: 17 April 2015). General patient characteristics as well as imaging data were collected and compiled in an anonymized data sheet.

### 2.2. Radiopharmaceutical and Imaging Protocol

Whole-body bone scintigraphy was performed after intravenous administration of ^99m^Tc-phosphonate (TECEOS, CIS bio GmbH, Berlin, Germany) at a mean of 19.3 ± 21.3 days prior to ^223^Ra therapy at the Department of Nuclear Medicine, LMU Munich. Images were acquired in planar imaging mode at a mean of 3 h after injection using a Siemens Symbia Intevo 16/6/2 2014 (Siemens Healthcare, Erlangen, Germany). Additionally, SPECT/CT images were performed (256 × 1024 matrix; scanning speed 15 cm/min).

### 2.3. Image Analysis

The aBSI was determined automatically by using EXINI Diagnostics AB (Lund, Sweden). The response to treatment was analyzed by investigating changes in aBSI as well as osseous total tumor volume (TTV) and SUV_max_ on SPECT/CT. A dedicated software package was used (Hermes Hybrid Viewer, version 2.0; Hermes Medical Solutions, Stockholm, Sweden).

### 2.4. ^223^Ra Therapy

^223^Ra dichloride (Xofigo^®^, Bayer AG, Leverkusen, Germany) was from Bayer. A mean dose of 4.3 ± 0.8 MBq per cycle was administered intravenously as described elsewhere [3,12]. The therapy was conducted in the outpatient department of the Department of Nuclear Medicine, LMU Munich. Patients underwent ^223^Ra therapy every three weeks for a total of three or six cycles. After three cycles, treatment was either continued up to six cycles or paused, depending on clinical assessment, tolerability, evidence of disease progression, or patient performance status. After three cycles, all patients underwent a bone scan to assess response. For those who continued and completed six cycles of ^223^Ra therapy, an additional bone scan was performed after the sixth cycle. Changes between baseline parameters and those obtained after either three or six therapy cycles—depending on the total number of cycles administered—were systematically analyzed.

Patients underwent laboratory analyses of hemoglobin level, white blood cell (WBC) counts, platelets, neutrophils, prostate-specific antigen (PSA), and alkaline phosphatase (AP) prior to every therapy cycle as well as 6–8 weeks following the last cycle.

### 2.5. Statistical Analysis

Data analysis was performed using Microsoft Excel (Excel 2019, Microsoft, Redmond, WA, USA) and GraphPad Prism (Version 9.5.0 (730)). The descriptive statistics are displayed as mean ± standard deviation (STD). Shapiro–Wilk test was used to assess data normality before applying parametric or non-parametric tests. Group comparisons were performed using a parametric or non-parametric unpaired *t*-test. Correlation analyses were conducted using Spearman or Pearson correlation analyses. Survival data were calculated using Kaplan–Meier-curves and Log rank test. A two-tailed *p*-value < 0.05 was considered statistically significant.

## 3. Results

### 3.1. Bone Scintigraphy, SPECT/CT and Baseline Lab Analysis

In total, 49 patients at a mean age of 70 ± 9 years diagnosed with mCRPC and extensive osseous tumor load on BS were included.

The mean baseline aBSI was 2.9 ± 3.4%, the mean TTV was 237.2 ± 217.1 mL, and the mean SUV_max_ was 878.7 ± 517.4. The baseline PSA levels were 99.1 ± 273.1 ng/mL, and the AP levels were 173.6 ± 207.0 U/L. The aBSI, the mean total tumor volume (r = 0.629; r^2^ = 0.396; *p* < 0.001), and the mean SUV_max_ (r = 0.301; r^2^ = 0.091; *p* = 0.035) correlated significantly (Table 1).

### 3.2. Changes in Clinical and Imaging Parameters Following ^223^Ra Therapy

Overall, 7 out of 49 (14.3%) patients underwent three cycles, while 42 out of 49 (85.7%) completed six cycles of ^223^Ra therapy.

**Figure 1:**
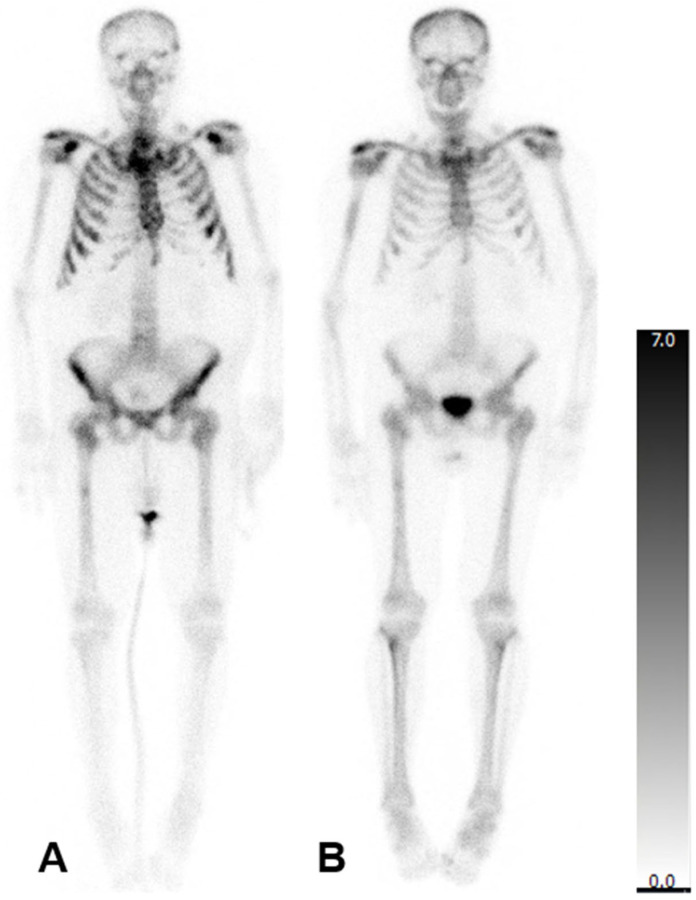
Bone scintigraphy of a 77-year-old man with mCRPC. aBSI prior to ^223^Ra therapy was 7.5%, TTV 1112.20 mL and SUV_max_ 388 (**A**). After three cycles of ^223^Ra therapy, aBSI was 6.7%, TTV was 97.03 mL, and SUV_max_ was 293 (**B**).

In patients who underwent three cycles of treatment, there was no statistically significant change in the mean aBSI (6.7 ± 5.2%; before: 4.5 ± 3.8%; *p* = 0.369), mean TTV (353.2 ± 369.2 mL; before: 356.1 ± 382.9 mL; *p* = 0.902), or mean SUV_max_ (562.3 ± 156.2; before: 801.7 ± 379.3; *p* = 0.149). The imaging of a representative patient case is presented in Figure 1.

Changes in aBSI were not significantly correlated with changes in TTV (r = 0.559; r^2^ = 0.312; *p* = 0.206) or SUV_max_ (r = −0.400; r^2^ = 0.160; *p* = 0.368).

The PSA level (953.4 ± 1884.2 ng/mL; before: 383.8 ± 673.4 ng/mL; *p* = 0.731) and the AP level (220.6 ± 172.7 U/L; before: 244.4 ± 241.0 U/L; *p* = 0.949) did not change significantly.

Considering the side effects of a higher grade (CTCAE ≥ 3), lymphopenia was seen in three patients (CTCAE grade 3), whereas anemia (CTCAE grade 3) and thrombopenia (CTCAE grade 4) were present in one patient each.

**Figure 2:**
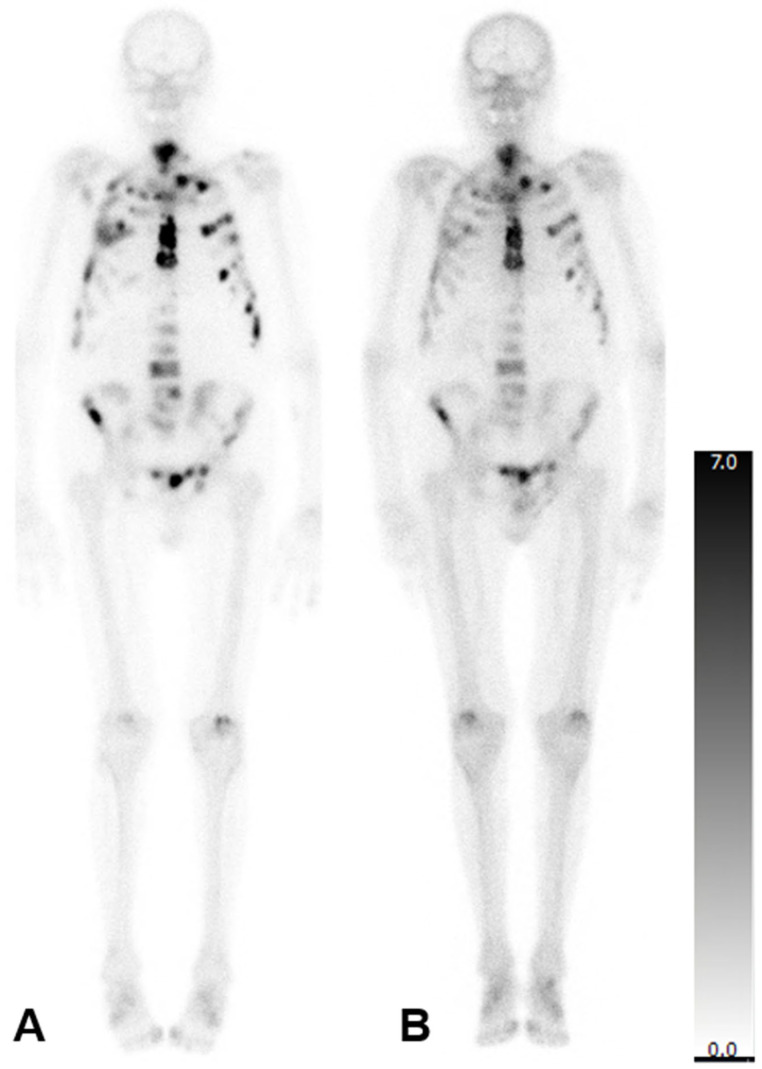
Bone scintigraphy of an 83-year-old man with mCRPC. aBSI prior to ^223^Ra therapy was 7.8%, TTV was 549.32 mL, and SUV_max_ was 1500 (**A**). After six cycles of ^223^Ra therapy aBSI was 4.1%, TTV was 368.05 mL, and SUV_max_ was 1284 (**B**).

In patients who were administered six cycles, there was no significant change in the mean aBSI (2.6 ± 4.3%; before: 2.6 ± 3.3%; *p* = 0.247) or the mean TTV (208.8 ± 185.2 mL; before: 217.4 ± 175.8 mL; *p* = 0.784). However, the mean SUV_max_ reduced significantly (548.1 ± 342.2; before: 891.6 ± 539.6; *p* = 0.001). A representative patient case is illustrated in Figure 2.

Changes in aBSI were significantly correlated with changes in TTV (r = 0.360; r^2^ = 0.130; *p* = 0.019), but not with changes in SUV_max_ (r = 0.132; r^2^ = 0.017; *p* = 0.405).

Additionally, the PSA level did not change significantly (73.5 ± 110.0 ng/mL; before: 51.7 ± 69.5 ng/mL; *p* = 0.656), whereas the AP level reduced significantly (101.3 ± 91.1 U/L; before: 161.6 ± 201.5 U/L; *p* = 0.010).

### 3.3. Overall Survival

In 5 out of 7 patients that underwent three cycles and in 17/42 patients that underwent six cycles, OS data were available. The mean OS was 809 ± 831 days, with 905 ± 896 days for patients undergoing six cycles and 484 ± 490 days for patients undergoing three cycles.

Log rank test showed no significant difference for patients undergoing six cycles of therapy when investigating the OS for patients with an aBSI above the mean of 2.6% and ≤2.6% (median OS > 2.6%: 431 days; median OS ≤ 2.6% 814 days; χ^2^ = 1.823, *p* = 0.177).

**Figure 3:**
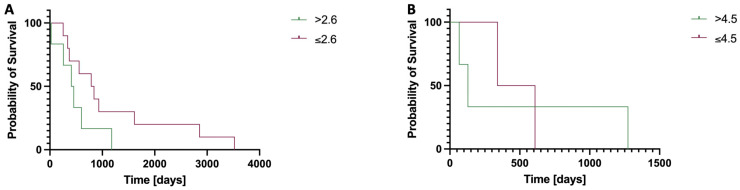
Kaplan–Meier curves comparing the overall survival (OS) in patients stratified by baseline aBSI values above or below the group mean, following six (**A**) or three (**B**) cycles of ^223^Ra therapy. (**A**) Median OS > 2.6%: 431 days; median OS ≤ 2.6% 814 days; although a trend toward improved survival was observed in patients with a lower aBSI, this difference did not reach statistical significance (χ^2^ = 1.823, *p* = 0.177). (**B**) Median OS > 4.5%: 129 days; median OS ≤ 4.5% 475 days; however, this difference was also not statistically significant (χ^2^ = 0.308, *p* = 0.579).

There was also no significant difference for patients undergoing three cycles of therapy when investigating the OS for patients with an aBSI above the mean of 4.5% and ≤4.5% (median OS > 4.5%: 129 days; median OS ≤ 4.5% 475 days; χ^2^ = 0.308, *p* = 0.579; Figure 3).

## 4. Discussion

With this study, we aimed to investigate the prognostic value of aBSI to assess the response to ^223^Ra therapy in patients undergoing three or six cycles.

After completing three or six cycles of ^223^Ra therapy, no significant change in mean aBSI was observed in either group. While TTV remained stable regardless of treatment duration, a significant reduction in mean SUV_max_ was seen after six cycles but not after three. This suggests that the therapeutic impact on tumor metabolism becomes more evident with prolonged treatment.

Correlation analyses showed a significant association between changes in aBSI and TTV exclusively after six cycles of treatment. In contrast, no significant correlations were found between changes in either aBSI and TTV after three cycles or between changes in SUV_max_ and aBSI at any time point.

In both treatment groups, PSA levels increased during the initial treatment phase, suggesting the presence of a PSA flare phenomenon [13]. This phenomenon describes a transient rise in PSA levels shortly after treatment initiation, which may not accurately reflect true disease progression but rather a biological response to therapy. The temporary rise in PSA can result in apparent discrepancy between biochemical markers (e.g., PSA) and imaging findings (e.g., TTV) despite stable or even regressing tumor burden, as observed in this study (stable TTV). The PSA flare phenomenon was initially reported in a case report and has since been documented in larger cohorts, particularly in the context of androgen deprivation therapy and other systemic treatments [13,14,15]. Mechanistically, the flare is thought to be caused by increased tumor cell lysis or immune-mediated effects, leading to the release of PSA into the bloodstream [16,17]. Importantly, some studies have found that PSA flares do not negatively impact outcomes and may even be associated with improved prognosis, reflecting effective tumor response despite the rise in transient PSA [14,16,18,19]. However, the flare also complicates early treatment assessment, as rising PSA levels might be misinterpreted as progression, potentially influencing clinical decisions. In contrast, AP levels showed a notable decline only after the completion of six cycles. However, previous studies indicate that alterations of AP levels are not associated with a prolonged overall survival [20]. Given the complex dynamics of PSA and AP levels, additional studies investigating changes in biomarkers and imaging findings are needed to better understand the flare phenomenon. Additionally, our data showed that treatment with ^223^Ra did not result in a significant reduction in TTV, indicating a stable disease rather than tumor regression.

Baseline aBSI was lower in patients who completed six cycles of treatment (mean aBSI: 2.6%) compared to those who received only three cycles (mean aBSI: 4.5%). Previous studies have demonstrated that a lower baseline aBSI is associated with more favorable outcomes and prolonged OS in patients with mCRPC [21,22,23]. However, in our cohort, no significant difference in OS was observed when comparing patients with an aBSI above versus below the respective group mean. This suggests that although the baseline aBSI reflects overall disease burden, its prognostic utility for predicting overall survival may be limited in this setting. The discrepancy between our findings and those reported in previous studies [9,10,11] may be attributed to differences in baseline patient characteristics or the small, heterogeneous nature of our cohort, which could have influenced the observed outcomes. Nevertheless, aBSI evaluation may serve as a practical and time-efficient method for estimating osseous tumor burden, especially when compared to the more complex and labor-intensive assessment of TTV. In our analysis, neither aBSI nor TTV showed significant changes following ^223^Ra therapy, irrespective of whether three or six cycles were administered. Although quantitative changes in these two parameters did not significantly correlate, their respective absolute values consistently reflected stable disease burden during treatment. This suggests that both metrics offer a comparable overall assessment of treatment response, despite reflecting different biological aspects of tumor progression.

In this study, adverse events classified as grade 3 or higher according to the CTCAE criteria were observed in five patients (10.2%). This incidence falls within an acceptable range and aligns with the findings of comparable studies [8,24,25]. It has been demonstrated that patients with pre-existing impaired hematopoiesis are particularly vulnerable to developing high-grade hepatotoxicity [26].

This study is limited primarily because of its retrospective nature and small sample size (*n* = 49). Its retrospective design led to an inherent imbalance between the treatment groups, with notably fewer patients receiving only three cycles. This limitation reduces statistical power and affects the robustness of survival analyses, which limits generalizability and necessiates the inclusion of larger cohorts and long-term follow-ups to validate the findings. The study is intended as a foundational exploration to inform and guide future prospective trials with more balanced cohorts. Additionally, the cohort of patients was heterogeneous due to the administration of various pretreatments (e.g., chemotherapy, ADT, radiation), which may have influenced the observed outcomes and responses. Long-term data regarding safety and outcome are needed, and the data of the REASSURE study are awaited [27]. In patients whose treatment was discontinued after three cycles—due to toxicity, disease progression, or poor clinical performance—it is likely that a more advanced or aggressive disease course was already present at baseline. This introduces a relevant selection bias that may have influenced both the decision to limit treatment duration and the observed clinical outcomes. In order to ensure methodological consistency and valid comparability of the imaging data, all imaging procedures were performed using the same scanners and identical reconstruction protocols.

Looking ahead, while the conventional use of BS for monitoring response to ^223^Ra therapy is well-established, the potential advantages of emerging imaging techniques, especially PSMA-PET/CT, should not be overlooked [28,29]. PSMA-PET targets the prostate-specific membrane antigen, providing a more detailed assessment of tumor metabolism [30]. Its superior sensitivity and specificity allow for a more accurate evaluation of metastatic burden and therapeutic response at the molecular level. As these technologies continue to evolve, they may become more suitable for monitoring the efficacy of ^223^Ra therapy, potentially improving patient management and outcome prediction in mCRPC, as suggested by Shagera et al. [28]. In parallel, novel treatment strategies are being developed to optimize treatment sequences in mCRPC. For instance, the RALU study investigated the outcomes of lutetium radioligand therapy following ^223^Ra therapy [27].

In light of the retrospectively collected dataset, we recognize that significant advancements have since occurred, including wider adoption of PSMA-PET imaging and increased use of PSMA-targeted radioligand therapies. However, bone scintigraphy remains widely used, especially in settings where PET is not available, and PSMA therapy is not yet universally accessible.

## 5. Conclusions

In conclusion, while changes in the aBSI did not significantly correlate with changes in TTV, both parameters remained stable during therapy. Although aBSI did not demonstrate prognostic value for overall survival in this cohort, it remains a practicable and readily applicable surrogate for assessing osseous tumor burden, particularly in comparison to TTV. Given the limited sample size, the findings of this study should be interpreted with caution and require further studies in larger, prospective cohorts to clarify the potential role of aBSI in treatment monitoring and decision-making.

## Figures and Tables

**Table 1:** Patient characteristics.

	Age	Baseline aBSI [%]	Baseline TTV [mL]	Baseline SUV_max_	End aBSI [%]	End TTV [mL]	End SUV_max_
Mean	70	2.9	237.2	878.7	3.2	229.4	550.2
STD	9	3.4	217.1	517.4	4.6	221.3	321.1
Mean_6cycles_		2.6	217.4	891.6	2.6	208.8	548.1
STD_6cycles_		3.3	175.8	539.6	4.3	185.2	342.2
Mean_3cycles_		4.5	356.1	801.7	6.7	353.2	562.3
STD_3cycles_		3.8	382.9	379.3	5.2	369.2	156.2

aBSI—automated bone scan index; STD—standard deviation; TTV—total tumor volume.

## Data Availability

The datasets used and/or analyzed during the current study are available from the corresponding author on reasonable request.

## Abbreviations

The following abbreviations are used in this manuscript:

aBSI	Automated Bone Scan Index
ADT	Androgen deprivation therapy
AP	Alkaline phosphatase
BS	Bone Scan
CR	Complete response
mCRPC	Metastatic castration-resistant prostate cancer
PD	Progressive disease
PR	Partial Response
PSA	Prostate-specific antigen
Ra	Radium
STD	Standard deviation
SD	Stable disease
TTV	Total tumor volume
WBC	White blood cell
